# Effects of Physical Exercise Input on the Exercise Adherence of College Students: The Chain Mediating Role of Sports Emotional Intelligence and Exercise Self-Efficacy

**DOI:** 10.3390/jintelligence12100094

**Published:** 2024-09-26

**Authors:** Dongzhen An, Jianhua Pan, Feng Ran, Donghuan Bai, Jia Zhang

**Affiliations:** 1 School of Sport and Recreation, Sichuan Tourism University, Chengdu 610100, China; 2 School of Physical Education, Chongqing University, Chongqing 400001, China; 3 School of Physical Education, Huaibei Normal University, Huaibei 235000, China

**Keywords:** physical exercise input, sports emotional intelligence, sports self-efficacy, exercise adherence, college student

## Abstract

Objective: The aims of this study were to investigate the effects and mechanisms of physical exercise input, sports emotional intelligence, and sports self-efficacy on exercise adherence, and to examine the chain-mediating role of sports emotional intelligence→sports self-efficacy. Methods: The Physical Exercise Input Scale, Exercise Adherence Scale, Sports Emotional Intelligence Scale, and Sports Self-Efficacy Scale were used to investigate 1390 college students in three universities in the Henan Province. Results: (1) Physical exercise input was a significant positive predictor of exercise adherence (β = 0.29, t = 5.78, *p* < 0.001); (2) sports emotional intelligence and sports self-efficacy mediated the relationship between physical exercise input and exercise adherence; (3) physical exercise input influenced exercise adherence through the separate mediating role of sports emotional intelligence (β = 0.10, t = 5.98, *p* < 0.001), the separate mediating role of sports self-efficacy (β = 0.13, t = 2.64, *p* < 0.01), and the chain mediating role of sports emotional intelligence→sports self-efficacy (β = 0.09, t = 2.80, *p* < 0.01). Conclusions: (1) Physical exercise input can positively predict the level of sports emotional intelligence and sports self-efficacy of college students; (2) Physical exercise input can not only directly influence college students’ exercise adherence but can also indirectly influence it through sports emotional intelligence or sports self-efficacy levels alone, as well as through the chain mediation of the two.

## 1. Introduction

College students’ physical fitness has received increasing attention in recent years, and improving their health is a topic of growing interest in academic research (Yin et al. 2012; Dong et al. 2022). The physical health status of college students has been declining year by year, and studies have found that poor lifestyles characterized by insufficient physical activity, sedentary behavior, sleep deprivation, etc., are essential factors that lead to obesity and decreased cardiorespiratory endurance among this group (Nelson et al. 2008; Al-Tammemi et al. 2020). For instance, research has shown that prolonged sedentary behavior is closely linked to an increased risk of obesity, cardiovascular diseases, and mental health issues such as anxiety and depression (Nogueira et al. 2018; Lavie et al. 2019). Furthermore, insufficient physical activity has been associated with diminished cardiorespiratory fitness and overall health deterioration (Myers et al. 2015). Chronic sleep deprivation has also been proven to exacerbate these issues, further compromising the physical and mental well-being of college students (Moussa-Chamari et al. 2024). Therefore, how college students can effectively improve their physical and mental health status has become an essential aspect of researchers’ attention.

Against this background of health problems, studying the factors influencing college students’ exercise adherence is of great practical value and theoretical value. Physical exercise is widely recognized as an effective means of health promotion and plays a crucial role in forming and consolidating a healthy lifestyle. Regular physical activity is associated with numerous physical benefits, including improved cardiovascular health (Warburton et al. 2006), enhanced muscular strength (Liu and Latham 2009), and better weight management (Jakicic and Otto 2005). Additionally, regular exercise has been shown to provide psychological benefits, such as reducing symptoms of anxiety and depression (Stathopoulou et al. 2006), enhancing mood (Reed and Buck 2009), and improving overall mental well-being (Penedo and Dahn 2005). However, these benefits can only be realized if physical exercise is performed consistently over time, highlighting the importance of exercise adherence among individuals, particularly college students, who are in a critical stage for developing lifelong habits. However, nearly 50% of exercise participants drop out in the first three to six months (Tremblay et al. 2011; Dong 2017). Studies have shown that college students have low weekly participation in short duration physical activity, and even drop out of physical activity at the end of their physical education (Hao and Yang 2022). Therefore, it is vital to explore the psychological mechanisms of college students’ exercise adherence and develop effective strategies for maintaining and promoting exercise behaviors so that they can devote themselves to long-term, regular physical exercise.

Exercise adherence refers to the regular participation in and persistence or continuity of physical exercise, including frequency, intensity, and duration (Lee 2020). It also refers to the psychological aspect of an individual’s long-term, active participation in regular exercise activities (Rodrigues et al. 2020). It reflects not only the actual exercise behavior of the participant and its consistency but also, more importantly, their psychological characteristics during exercise (Burnet et al. 2020). Regular adherence to physical activity is essential in helping individuals plan, implement, and develop lifelong physical activity habits (Song and Zhang 2022). Among the relevant psychological mechanism models of exercise behavior, health belief theory, the theory of planned behavior, sport commitment theory, and the cognitive decision-making model of exercise adherence are often used to explain and predict exercise adherence behaviors, which provide a theoretical framework for the study of exercise adherence. Studies have found that motivation to participate (Chen et al. 2022), emotion regulation (He et al. 2022), commitment (Zhu and Dong 2016), and sense of efficacy (Yan et al. 2019) have an impact on exercise adherence. Among them, the cognitive decision model of exercise adherence, with the introduction of emotional experience, could better predict college students’ exercise adherence behaviors (Wu 2019). The present study is based on cognitive behavioral theory and the cognitive decision-making model of exercise adherence to explain and predict the impact of physical exercise input on college students’ exercise adherence.

Studies have shown that exercise adherence behavior depends on the level of physical exercise input and reflects a need or determination to persist in physical activity (Park et al. 2021). Physical exercise input refers to the positive, active, long-lasting, immersive psychological state, and pleasurable experience that individuals have during physical activity, manifested in the individual’s correct understanding of their physical activity behaviors and role identity (Wang et al. 2019; Gao et al. 2021). Meanwhile, it has been shown that physical exercise can be positive and influential in promoting exercise adherence. It ensures intrinsic exercise satisfaction, especially in improving positive emotional states and quality of life, which may further influence lifelong physical activity habits (Li et al. 2020). However, even though physical activity must be performed regularly to maximize its effects, most people drop out within a few months of its initiation. Therefore, maintaining exercise adherence and identifying the factors influencing it are topics that need to be addressed. Accordingly, hypothesis H1 is proposed: Physical exercise input positively predicts exercise adherence.

In the process of physical exercise, the emotional experience and self-efficacy of college students will impact their exercise behavior and the resulting effects, such as improvements in mental health (Zhang et al. 2024a), motivation (Garn and Simonton 2022), physical fitness (Joseph et al. 2014), or overall happiness (Khazaee-Pool et al. 2015), which are essential factors affecting their adherence (Javelle et al. 2023; Fu et al. 2023). In this study, we constructed a dual-mediation model of college students’ commitment and adherence to physical activity through two mediating variables, namely, sports emotional intelligence and self-efficacy. This study analyzed the relationship between the two to provide theoretical and practical references for promoting the physical health of college students, providing better guidance for college students’ physical activity, and supporting the development of school sports.

One of the concerns of this study regarding mediating mechanisms is the mediating effect of emotional intelligence in sports. Over the years, researchers have attempted to reveal how the emotional aspects of sports influence participation in sports behaviors, which have contributed significantly to the development of theoretical knowledge. For instance, the meta-emotional theory has been used in sports research; it integrates the cognitive processes, functions, and capacities of emotions. In sports, input emotional information undergoes three-stage cognitive processing (recognizing/understanding, managing/regulating, and utilizing/facilitating) for optimal motivational and behavioral output (Yoo 2010). It has been shown that sports participants experience a wide range of emotions during physical activity and that sports emotional intelligence can determine their sports behavioral tendencies and physical activity performance based on the characteristics of the emotions experienced and their regulation or control (Laborde et al. 2016; Zhao et al. 2016). Sports participants with high emotional intelligence will have meta-emotional functioning, recognizing, and regulating the unique emotions that arise from physical activity and using them positively for individual and team performance (Lee et al. 2020).

Sports emotional intelligence refers to the ability of individuals to effectively perceive, utilize, understand, and manage emotions in a sports environment. This concept extends the general understanding of emotional intelligence to the specific domain of sports and exercise, focusing on how emotional skills affect sports behavior (Levillain et al. 2022; Zhang et al. 2022). Physical exercise fulfills an internal need to experience immersion in physical activity. Observing the changes in positive emotions that occur physically and mentally after exercise can generate a sustained emotional identification with exercise (Daniels et al. 2021). Since emotional states influence exercise participation and persistence, the positive, affirmative emotions brought about through exercise have a positive effect (Inhyung 2022; Zou et al. 2024). According to reinforcement theory, it is known that the positive reinforcement effect of physical activity can promote positive emotions. Moreover, positive emotions can help individuals build lasting psychological resources, which in turn increase their level of emotional competence (Hong and Liu 2011; Gordan and Krishanan 2014).

Research has shown that sports emotional intelligence has a direct effect on physical activity performance and exercise adherence and constructs positive and persistent perceptions of physical activity through the perception and management of the positive emotional factors that are associated with exercise (Laborde et al. 2018). There is growing evidence that sports emotional intelligence plays a vital role in physical activity, so understanding this concept in the context of sports is particularly important for empirical interventions to improve exercise adherence. In summary, college students’ physical exercise input may influence exercise adherence through sports emotional intelligence. In this way, hypothesis H2 is proposed: Sports emotional intelligence mediates the relationship between college students’ physical exercise input and exercise adherence.

Another mediating variable of interest in this study is exercise self-efficacy. This refers to an individual’s confidence or belief in their ability to perform physical activity consistently and regularly to achieve a behavioral outcome; it is more likely to be a prognosticator of exercise competence (González et al. 2010). Research has shown that engagement in physical activity has a positive effect on exercise self-efficacy, which can have an impact on the persistence of physical activity and lead to a sense of satisfaction and positive emotions, and is highly associated with adherence to physical activity (Ganz et al. 2021; Wu et al. 2022). Exercise self-efficacy provides individuals with more stable emotional and behavioral motivation, significantly impacts their physical activity behavior, and is an essential influence on the partial or independent completion of established physical activities (McAuley et al. 2003; Neupert et al. 2009). Individuals with high levels of self-efficacy experience positive changes in their behavior, such as being more active, focused, and immersed, and can influence their own physical activity behavior and exercise adherence activities (Neace et al. 2022; Wiedenman et al. 2023). Individuals with high levels of exercise self-efficacy will have high expectations for their behaviors and tend to sustain physical activity behaviors over time (Zou et al. 2023). In this way, hypothesis H3 is proposed: Exercise self-efficacy mediates the relationship between college students’ physical exercise input and exercise adherence.

Cognitive behavioral theory states that cognition is the coordination between cognition, emotion, and behavior (Hagger and Hamilton 2021). The perception of physiological responses affects one’s emotions and cognition (Beauchamp et al. 2019). So, how does sports emotional intelligence affect exercise adherence? Exercise self-efficacy is the strength of an individual’s belief that they can successfully perform and complete an exercise task (Neace et al. 2022), i.e., it is a form of belief and self-confidence among exercise participants (Wiedenman et al. 2023). Research has shown a positive predictive effect of emotional intelligence on exercise self-efficacy (Annesi and Powell 2023). College students with high sports emotional intelligence will be more active in collecting information related to physical activity, learning necessary sports skills, and enhancing their self-confidence in performing physical activity behaviors, ultimately leading to higher-than-average sports self-efficacy (Shenaar-Golan et al. 2020; Bañoset et al. 2023). According to self-efficacy theory, self-efficacy can effectively predict individual behavior and is the psychological motivation for individuals to sustain self-regulation (Bandura 1997). Exercise self-efficacy, as a specific manifestation of self-efficacy in the context of physical activity, significantly affects an individual’s willingness and intention to engage in physical activity. Several empirical studies have shown that exercise self-efficacy has positive predictive validity for exercise adherence. Therefore, the effect of sports emotional intelligence on exercise adherence may be realized through exercise self-efficacy (Xu and Liu 2022; Zou et al. 2023).

Physical exercise input, as an essential indicator of a positive mental state, is a state of physical and psychological vitality that results in a strong interaction between an individual’s cognition and emotion related to physical exercise behavior (Luo 2022), which is conducive to improvements in exercise self-efficacy. Research has shown that exercise self-efficacy is higher when the experience of positive emotions during exercise is higher (Wang et al. 2020). Positive emotions can enhance self-efficacy through successful experiences or healthy living in general, and emotions related to exercise such as fun, pride, vigor, and achievement have been found to enhance self-efficacy in older adults (Jiang et al. 2018), which provides insights for research related to exercise-related emotions and efficacy in college students. From this, it is reasonable to hypothesize that, when an individual’s level of physical exercise is high, the related positive emotions result in positive cognition in terms of the perception and management of the individual’s emotional intelligence. Through this, exercise self-efficacy is further enhanced and behavioral decisions are made through situational attribution, ultimately influencing exercise adherence. Thus, hypothesis H4 is proposed: Exercise self-efficacy mediates between sports emotional intelligence and exercise adherence in college students.

## 2. Materials and Methods

### 2.1. Participants

In this study, an online survey system was employed from September to October 2023. Three colleges and universities in the Henan Province were selected, and the cluster sampling method was used to distribute the questionnaires by class. The Sojump was used to design the questionnaire and generate a network link that was shared in classrooms at the three universities via WeChat (Tencent Holdings Ltd., Shenzhen, China) or QQ group (Tencent Holdings Ltd., Shenzhen, China). The minimum sample size required for performing a correlation analysis was calculated to be 301 (α error probability 0.05, test validity 80%) using G*Power3.1 software. Therefore, the number of analytical samples retained satisfies the recommended sample size for questionnaire surveys.

To improve the authenticity of the data, the actual completion time and quality of the online questionnaires were examined, and after the exclusion of invalid questionnaires (such as answering the questionnaire in less than 120s and regular answers), a total of 1553 questionnaires were distributed, and 1390 valid questionnaires were recovered (validity rate = 89.5%). The sample comprised 758 males (54.5%) and 632 females (45.5%), including 452 freshmen (32.5%), 558 sophomores (40.1%), and 380 juniors and above (27.4%); 456 people (32.8%) had participated in sports and exercise activities for less than three months, 274 people (19.7%) had participated in sports and exercise activities for 3–6 months, 226 people (16.3%) had participated in sports and exercise activities for 6–12 months, 244 (17.6%) for 1–2 years, and 190 (13.6%) for more than 2 years. The physical exercises performed by the college students in this study included basketball, football, volleyball, running, table tennis, badminton, tennis, jumping rope, and fitness.

This study was approved by the Ethics Committee of the School of Education at Sichuan Tourism University. All of the procedures were performed in accordance with the Declaration of Helsinki and relevant policies in China. All participants agreed to participate voluntarily, with informed consent obtained when they filled in the survey and were able to withdraw from the study at any time. The questionnaire was designed and applied to ensure the anonymity of participants. The data were confidential, and participation was anonymous without any potential risk to the integrity of the subjects.

### 2.2. Measurement

#### 2.2.1. Physical Exercise Input Scale

The Physical Exercise Input Scale was developed by Dong and Mao (2018). Dong and Mao (2018) reported the reliability of their scale using Cronbach’s α coefficient of 0.942, with a split-half reliability of 0.889. They also supported its validity by the correlations shown by this measure with other measures of physical exercise input such as exercise commitment and subjective experience, and its ability to predict physical exercise habits. The scale includes four dimensions with 20 question items: vigor persistence (e.g., *I can maintain sufficient vigour and full enthusiasm during physical exercise.*), concentration satisfaction (e.g., *I am immersed in physical activity.*), value perception (e.g., *I am in agreement with my ability to exercise.*), and participation autonomy (e.g., *I am able to freely choose my own form of physical activity.*). A five-point scale was used, ranging from not conforming (1 point) to fully conforming (5 points), with higher scores indicating higher commitment to physical activity. In this study, Cronbach’s alpha coefficient of the scale in this study was 0.85. The validated factor analysis model fit indices were RMSEA = 0.06, CFI = 0.92, TLI = 0.91, and SRMR = 0.07, which indicated that the reliability index of the scale was good.

#### 2.2.2. Exercise Adherence Scale

The Exercise Adherence Scale which was developed and revised by Lee and Kim (2016), was used. Lee and Kim (2016) reported the reliability of their scale using Cronbach’s α coefficient of 0.863. They also supported its validity by the correlations shown by this measure with other measures of exercise adherence, such as satisfaction of class and fun factor. The scale consists of 1 dimension with 6 question items (e.g., *I’m willing to make regular time for consistent physical activity.*). A five-point scale ranged from complete noncompliance (1 point) to complete compliance (5 points), with higher scores indicating higher physical activity adherence. In this study, Cronbach’s alpha coefficient for the scale in this study was 0.82. The validated factor analysis model fit indices were RMSEA = 0.07, CFI = 0.93, TLI = 0.95, and SRMR = 0.05, which indicated that the reliability indices of the scale were good.

#### 2.2.3. Sports Emotional Intelligence Scale

The study used a 14-item Sports Emotional Intelligence Scale (SEIS) developed by Zhang et al. (2022) to measure sports emotional intelligence. Zhang et al. (2022) reported the reliability of their scale using Cronbach’s α coefficient of 0.905, and the split-half reliability of 0.891. They also supported its validity by the correlations shown by this measure with other measures of sports emotional intelligence, such as exercise commitment and exercise adherence. The scale includes four dimensions: emotional evaluation of others (e.g., *I can usually guess my friends’ emotions from their behavior.*), self-emotion management (e.g., *During physical exercise, I often know why I feel happy or unhappy.*), emotional use (e.g., *When physical exercise, I know the reasons for the emotional changes I’ve experienced.*), and social skills (e.g., *I prefer to share my emotions with my peers or coaches when physical exercise.*). A five-point scale was used, ranging from not at all (1 point) to fully compliant (5 points), with higher scores indicating higher levels of sports emotional intelligence among college students. In this study, Cronbach’s alpha coefficient for the scale in this study was 0.87. The validated factor analysis model fit indices were RMSEA = 0.07, CFI = 0.93, TLI = 0.95, and SRMR = 0.04, which indicated that the reliability indices of the scale were good.

#### 2.2.4. Exercise Self-Efficacy Scale

This study used 10 items from Kim (2015) to measure Exercise Self-Efficacy. Kim (2015) reported the reliability of their scale using the Cronbach’s α coefficient of 0.844. Kim also supported its validity by the correlations shown by this measure with other measures of exercise self-efficacy, such as exercise behaviors, and its ability to predict perceived health status. The scale consists of 1 dimension with 10 question items (e.g., *Even if I get tired during a physical exercise, I can still keep up with completing it.*). A five-point scale was used, ranging from non-compliant (1 point) to fully compliant (5 points), with higher scores indicating higher levels of exercise self-efficacy. In this study, Cronbach’s alpha coefficient for the scale in this study was 0.82. The validated factor analysis model fit indices were RMSEA = 0.07, CFI = 0.97, TLI = 0.94, and SRMR = 0.03, which indicated that the scale had good reliability indicators.

### 2.3. Data Processing and Analysis

SPSS25.0 was used to conduct descriptive statistics, reliability and validity, standard deviation, and correlation analyses of the data for each variable. Mplus8.0 was used to conduct structural model analysis to test the mediating effects of sports emotional intelligence and exercise self-efficacy on the physical exercise input and exercise adherence of college students. The mediation effects of sports emotional intelligence and exercise self-efficacy in physical exercise input on exercise adherence were tested first. Then, the chain mediation effect of sports emotional intelligence and exercise self-efficacy was tested.

## 3. Results

### 3.1. Common Method Bias Test

The Harmon one-way method was used to test for common method bias. An exploratory factor analysis of all questionnaire questions revealed that the variance explained by the first factor without rotation was 23.5%, less than the critical criterion of 40%. It can be inferred that common method bias was insignificant in this study (Zhang et al. 2024b).

### 3.2. Correlations and Descriptive Statistics

The results of the correlation analysis of the variables showed (see Table 1) that there was a significant positive correlation between physical exercise input, sports emotional intelligence, exercise self-efficacy, and exercise adherence.

### 3.3. The Relationship between Physical Exercise Input and Exercise Adherence: A Chain Mediation Effect Test

**Figure 1:**
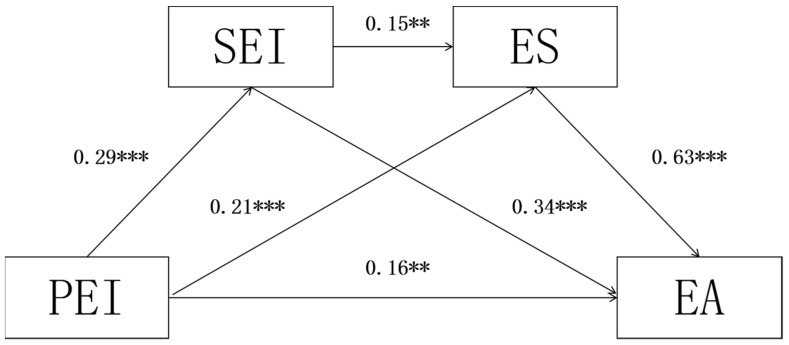
Chain mediation model of physical exercise input on exercise adherence. Note: *** and ** indicate 1% and 5% significance levels, respectively.

This study used structural equation modelling to test the mediating effects of sports emotional intelligence and exercise self-efficacy between physical exercise input and exercise adherence. First, the fit indices of the hypothesized model were assessed, and the results showed that the hypothesized model fit well (RMSEA = 0.04, TLI = 0.92, CFI = 0.94, NNFI = 0.02). Second, the mediating effects of sports emotional intelligence and exercise self-efficacy in the relationship between physical exercise input and exercise adherence were tested (see Table 2). The results of the test of chain mediation showed (see Figure 1) that physical exercise input positively predicted college students’ sports emotional intelligence (β = 0.29, t = 5.78, *p* < 0.001); both physical exercise input and sports emotional intelligence significantly positively predicted exercise self-efficacy (β = 0.21, t = 5.32, *p* < 0.001; β = 0.15, t = 4.86, *p* < 0.01); physical exercise input, exercise emotional intelligence, and exercise self-efficacy were also significant positive predictors of exercise adherence (β = 0.16, t = 4.96, *p* < 0.01; β = 0.34, t = 5.75, *p* < 0.001; β = 0.63, t = 6.89, *p* < 0.001).

A bias-corrected percentile bootstrap (5000 replicate samples) was used to test for mediating effects, as shown in Table 3. The 95% interval of the mediating effect of sports emotional intelligence on physical exercise input and college students’ exercise adherence did not contain 0, and the mediating effect was significant. Therefore, sports emotional intelligence mediates between physical exercise input and exercise adherence, and hypothesis 2 was verified. The 95% interval of the mediating effect of exercise self-efficacy on physical exercise input and exercise adherence did not contain 0, and the mediating effect was significant. Therefore, exercise self-efficacy mediates between physical exercise input and exercise adherence, and hypothesis 3 was proven. The 95% interval of the mediating effect of exercise self-efficacy on sports emotional intelligence and exercise adherence did not include 0, and the mediation effect was significant. Therefore, exercise self-efficacy mediates between sports emotional intelligence and exercise adherence; hypothesis 4 was proven. The 95% interval of the chained mediation effect of sports emotional intelligence and exercise self-efficacy on physical exercise input and exercise adherence did not contain 0, and the chained mediation effect was significant. Therefore, sports emotional intelligence and exercise self-efficacy act as a chain mediator between physical exercise input and exercise adherence.

## 4. Discussion

Based on cognitive behavioral theory, the cognitive decision theory model of exercise adherence, and related research results, this study explored and analyzed the relationship and mechanism of college students’ physical exercise input and exercise adherence. A significant positive correlation was found between physical exercise input and exercise adherence, verifying hypothesis H1. The results of the mediation model comprising sports emotional intelligence and exercise self-efficacy showed that these two factors partially mediated the relationship between physical exercise input and exercise adherence. There were three paths of mediation in the research model: mediation through sports emotional intelligence and exercise self-efficacy individually, and the chain mediation of sports emotional intelligence and exercise self-efficacy. These results verified hypotheses H2, H3, and H4.

### 4.1. The Effect of Physical Activity Input on Exercise Adherence

The cultivation of college students’ sports literacy and lifelong sports awareness is the center of sports work in colleges and universities (Li and Hu 2023). This study takes college students’ extracurricular physical exercise as a starting point to explore and study the mechanism of its role on exercise adherence. This study found that college students’ physical exercise input has a positive predictive effect on exercise adherence, consistent with the results of previous studies. Prior research has shown that physical exercise input is a rational psychological factor for college students to establish exercise habits, and their exercise adherence increases as cognitive and behavioral inputs increase through the mediating effect of psychological satisfaction (Bing et al. 2019). Physical exercise input is the satisfaction of participants’ desire to actively engage in physical activity, which promotes interest in these activities (Lee et al. 2015) and influences and increases the likelihood of sustained participation. Building on this, the present study introduced a chain mediation model between sports emotional intelligence and exercise self-efficacy, and obtained some meaningful findings.

### 4.2. Separate Mediating Roles of Emotional Intelligence and Sport Self-Efficacy

The results of this study indicated that college students’ physical exercise input could influence exercise adherence through the separate mediating roles of sports emotional intelligence and exercise self-efficacy, consistent with the results of previous studies. Prior studies have found that sports emotional intelligence mediates the relationship between physical exercise input and enthusiasm for exercise (So 2019), and exercise self-efficacy mediates the relationship between transactional leadership behavior and willingness for exercise adherence (Yan et al. 2019). Research has shown that high levels of physical exercise input enhance individual exercise performance and provide psychological benefits such as feelings of accomplishment, psychological satisfaction, self-efficacy, and positive mood changes (Jin et al. 2018; Mutz et al. 2021). In contrast, this psychological satisfaction is an intrinsic motivation for physical activity engagement (Lee and Moon 2017). These positive emotions are controlled and regulated by emotional intelligence to maintain a higher enthusiasm for participation in sports, which is the driving force for consistent participation in physical activity. Albert’s ABC theory of emotions states that things do not affect people; rather, people are affected by their perceptions of things (Wu 2019). Further validating the mediating role of sports emotional intelligence between physical exercise input and exercise adherence, the antecedents of things A (physical exercise input) will produce emotional and behavioral outcomes C (exercise adherence) through the evaluation and interpretation of positive emotions by B (sports emotional intelligence).

It has been shown that participants who engage in physical activity have a high perception of subjective health status. At the same time, there is a high correlation between subjective and actual health status (Baron-Epel and Kaplan 2001). Therefore, positive emotions such as psychological satisfaction and perception of physical activity behaviors resulting from physical exercise input can affect individuals’ exercise self-efficacy. Exercise self-efficacy is an essential concept in the model of behavioral change, as it is an action belief necessary for individuals to believe that they can achieve a specific outcome through physical activity, and it is closely related to changes in physical activity behavior (Fletcher and Banasik 2001). Further, this study also validates the cognitive behavioral theory, which states that an individual’s cognitive and life patterns are constructed and regulated by correctly interpreting external events and performing effective self-adaptation to construct and regulate behavioral goals. The higher the exercise self-efficacy, the more positive the perception of subjective health status and the more stable the behavioral goals for exercise adherence. Therefore, physical exercise input will impact exercise adherence in college students through the mediating role of exercise self-efficacy.

### 4.3. Chain Mediation of Motor Emotional Intelligence and Motor Self-Efficacy

In this study, we found that the chain mediation effects of sports emotional intelligence and exercise self-efficacy were significant in the relationship between physical exercise input and exercise adherence in college students. This not only indicated that sports emotional intelligence and exercise self-efficacy were vital in the mediation pathway of physical exercise affecting exercise adherence, but also explained college students’ insufficient and inconsistent physical exercise input. The broaden-and-build theory of positive emotions suggests that positive emotions can expand an individual’s attention, cognition, and behavior and construct and enhance individual persistence resources (Fredrickson 2011). A good physical exercise input experience can provide individuals with positive emotional experiences, help them adopt appropriate ways to cope with adverse situations and maintain healthy emotions, make their attitudes more positive, and enhance their exercise self-efficacy (Kim 2021). High sports emotional intelligence is a critical factor for individuals to manage and regulate their emotions, thus forming the influence pathway “physical exercise input → sports emotional intelligence → exercise self-efficacy”.

The present study also found that sports emotional intelligence indirectly affects exercise adherence through exercise self-efficacy, and individuals with higher sports emotional intelligence have higher exercise self-efficacy (Mei et al. 2019), providing empirical support for social cognitive theory. Sports emotional intelligence involves an individual’s self-perception and self-regulation, which are vital in developing exercise self-efficacy (Udayar et al. 2020). College students with high sports emotional intelligence can better understand different physical exercise inputs during physical activity, have better contextual attribution when dealing with events that may occur during physical activity, and have higher exercise self-efficacy. The present study found that exercise self-efficacy mediated the relationship between sports emotional intelligence and exercise adherence, i.e., college students with high sports emotional intelligence had high exercise self-efficacy, which enhanced their adherence to exercise.

Overall, this study proposed that the chain mediation model based on the theoretical foundation reveals the mechanism of college students’ physical exercise input on exercise adherence in a more in-depth way. College students’ physical exercise input affects exercise adherence both directly and indirectly through sports emotional intelligence and exercise self-efficacy, consistent with previous studies. The higher their physical exercise input, the easier it is for them to generate positive emotions, strengthen psychological resources, and improve sports emotional intelligence. Based on this, sports emotional intelligence can regulate and control positive emotions, resulting in increased psychological satisfaction and subjective health status, and thus enhancing exercise self-efficacy and improving exercise adherence.

## 5. Limitations

This study has several limitations that should be addressed. First, it only explored the relationship between physical exercise input and exercise adherence and the mediating mechanisms of sports emotional intelligence and exercise self-efficacy; future studies should consider other dimensions of physical exercise input or include more mediating and moderating variables. Second, this study used questionnaires and cross-sectional studies; future studies should further verify the causal relationship between variables using experimental and longitudinal methods. In addition, this study mainly focused on college students; caution should be taken when generalizing the findings to other groups.

## 6. Implication

This study has important theoretical and practical significance. In terms of theory, existing studies have explained the relationship between physical exercise input and exercise adherence more from the perspective of the theory of planned behavior or exercise commitment theory. This study introduces a cognitive decision-making model of exercise adherence, which argues that exercise behavior is affected not only by rational decision-making processes but also by irrational components. Among them, emotional experience in physical exercise is an important influence. This study reveals the critical role of sports emotional intelligence and self-efficacy in physical activity behavior, demonstrating the importance of emotion management and self-confidence enhancement in long-term exercise. In addition, this study provides a theoretical basis for further exploring the interactions of different psychological factors in exercise behavior and advances the understanding of the complexity and diversity of physical activity behaviors.

From a practical application perspective, the results of this study have important guiding significance for physical education and health promotion programs in higher education. College students’ physical health has been declining year by year, so research on exercise adherence in this population supports the critical role of school sports. The results of this study reflect the positive impact of physical exercise input on individual exercise adherence. By understanding the mediating roles of sports emotional intelligence and self-efficacy in exercise adherence, educators can develop well-targeted teaching methods to foster students’ emotional regulation and self-efficacy in physical activity and help them better cope with challenges and difficulties in exercise. School administrators can design more effective interventions and incentives to stimulate students’ interest in physical activity and enhance their adherence. Enhancing students’ emotional intelligence and self-efficacy in sports can not only improve their sports skills but also promote the development of physical and psychological health, which in turn will promote the formation of lifelong sports habits and lay a solid foundation for achieving the goal of health for all.

## 7. Conclusions

This study concluded that physical exercise input, sports emotional intelligence, and exercise self-efficacy had positive predictive effects on exercise adherence, with physical exercise input having a significant direct effect on exercise adherence, and sports emotional intelligence and exercise self-efficacy having a chained mediating effect between physical exercise input and exercise adherence.

## Figures and Tables

**Table 1:** Results of descriptive statistics and correlation analysis of each variable.

Variable	M	SD	PEI	SEI	ES	EA
PEI	3.83	0.54	1			
SEI	3.85	0.59	0.55 **	1		
ES	3.45	0.53	0.40 **	0.47 **	1	
EA	3.80	0.47	0.35 **	0.24 **	0.19 **	1

Note: N = 1390; ** indicates 5% level of significance; PEI: physical exercise input; SEI: sports emotional intelligence; ES: exercise self-efficacy; EA: exercise adherence.

**Table 2:** Regression analysis of the mediation model.

Regression Equation		Overall Fit		Significance of Regression Coefficients	
Outcome Variable	Predictor Variable	*R* ^2^	*F*	*β*	*t*
SEI	PEI	0.35	14.54	0.29	5.78 ***
ES	PEI	0.38	15.81	0.21	5.32 ***
	SEI			0.15	4.55 **
EA	PEI	0.36	14.89	0.16	4.96 **
	SEI			0.34	5.75 ***
	ES			0.63	6.89 ***

Note: *** and ** indicate 1% and 5% significance levels, respectively; PEI: physical exercise input; SEI: sports emotional intelligence; ES: exercise self-efficacy; EA: exercise adherence.

**Table 3:** Comparison table of indirect effect analysis.

Path	Point Estimate	Product of Coefficients			5000 Bootstrap 95% CI			
					Bias Corrected		Percentile	
		S.E	Z	P	Lower	Upper	Lower	Upper
PEI→SEI→EA	0.10	0.03	5.98	0.000	0.10	0.22	0.11	0.24
PEI→ES→EA	0.13	0.01	2.64	0.009	0.01	0.04	0.02	0.04
PEI→SEI→ES→EA	0.09	0.01	2.80	0.006	0.01	0.02	0.01	0.02
Total effect	0.32	0.04	5.36	0.000	0.12	0.25	0.13	0.28

## Data Availability

The data presented in this study are available on request from the corresponding author.
